# Desired Numbers of Children, Fertility Preferences and Related Factors among Couples Who Referred to Pre-Marriage Counseling in Alborz Province, Iran

**DOI:** 10.22074/ijfs.2017.5010

**Published:** 2017-09-03

**Authors:** Razieh Lotfi, Masoumeh Rajabi Naeeni, Nasrin Rezaei, Malihe Farid, Afsoon Tizvir

**Affiliations:** 1 Social Determinants of Health Research Center, Alborz University of Medical Sciences, Karaj, Iran; 2 Department of Midwifery and Reproductive Health, School of Nursing and Midwifery, Alborz University of Medical Sciences, Karaj, Iran; 3 Department of Midwifery and Reproductive Health, School of Nursing and Midwifery, Shahid Beheshti University of Medical Sciences, Tehran, Iran; 4 Alborz University of Medical Sciences, Karaj, Iran; 5 Department of Community Medicine, School of Medicine, Alborz University of Medical Sciences, Karaj, Iran; 6 Deputy of Chancellor for Health, Alborz University of Medical Sciences, Karaj, Iran

**Keywords:** Fertility, Fertility Preferences, Time to Pregnancy, Family Size

## Abstract

**Background:**

The Islamic Republic of Iran has experienced a dramatic decrease in fertility rates in the past three decades. One of the main issues in the field of fertility is the couple’s preferences and the desire to bear children. This study aimed to determine desired number of children, fertility preference, and related factors among people referring pre-marriage counseling to clarify their presumed behavior in case of fertility.

**Materials and Methods:**

This study was a descriptive analytic cross-sectional survey, conducted during 8 months. The participants were 300 couples came to pre-marriage counseling centers of two health centers of Karaj and asked to complete a 22 items questionnaire about of demographic characteristics, participants’ interest, preference about fertility, and economic situation.

**Results:**

Majority of the males were between the ages of 20-30 years (66.6%) while majority of the females were below 25 years of age (57%). About 17 percent of men and 22.3 percent of women stated that they want to have 1 child and equally 52.7 percent of men and 52.7 percent of women wanted to have 2 children. The only factor that contributed to the female participant’s decision for a desirable number of children was the number of siblings that they have. In male participants with an increasing age at marriage and aspiration for higher educational level, the time interval between marriage and the birth of the first child has increased. There was a convergence in desired number of children in male and female participants.

**Conclusion:**

Majority of the participants express their desire to have only one or two children in future but in considering the fact that what one desires does not always come into reality, the risk of reduced fertility is generally present in the community. Appropriate policies should be implemented in order to create a favorable environment for children.

## Introduction

Changes in fertility rates are seen throughout the world. The Islamic Republic of Iran has experienced a dramatic decrease in fertility rates in the past three decades. Population studies showed that total fertility rate (TFR) has decreased from 7.7 in 1966 to 6.3 in 1976. Years after the Islamic Revolution, there was a slight increase in fertility rate to about 7 in 1980 but with initiation of the country’s national family planning program, this figure has declined to 5.5 (1). The fertility rate in the Islamic Republic of Iran fell dramatically from around 7.0 births per woman in the early 1980s to 1.9 births per woman in 2006. The total fertility rate was below replacement level from 2006 onwards. Based on the report given by the world bank, Iran’s population growth rate will be below 1% by 2025 (2).

Also based on the 2006 census, the National Statistical Center of Iran has reported a TFR of 1.9. Postponing of childbearing is one of the reasons of fertility decline (3). Also childbearing has been postponed due to several reasons; advanced maternal age at marriage, higher education and the aim to secure economic stability before conception (4). In Iran, fertility rate is influenced by factors such as education, number of children, age of partner and age at first marriage while age and income are not considered important factors (5). It is the fact that, the preference of family size and women’s social background has great relevance. A study conducted in China showed that factors contributing in family’s preference for smaller family size include; young age, preference to reside in the city, and pursue higher education (6). Lower education was associated with more number of children.

In Japan, younger women desire to have fewer children while women in rural areas prefer to have more numbers of children than those residing in urban areas. The mean desired number of children was 2.55 which were significantly more than the mean actual number of children 1.77 in all generations (7). One of the most important issues in relation to fertility is the age of mother at the time of attempting conception. Results for study conducted in the United States of America to assess the attitudes and awareness of male and female students regarding conception, indicated that although 90% of these students showed desire to have children and highly valued parenting as an important part of their future; lack of understanding between age related fertility decline and the complications associated with higher maternal age and delayed conception proved to be extensively high (3). Due to Iran’s declining population, the government and policymakers have expressed great concern regarding the sharp decline and the negative growth in the next 40 years. Accordingly, policies on population control that have been implemented in Iran for 25 years have been abandoned and those policies encouraging birth in the country’s 6th development program are very much emphasized. It seems that socio-economic changes that have been shaped in Iran’s society would make it difficult to reverse the trend of the country’s fertility rate. Delayed fertility is related to factors including; economic, social, and cultural which can be affected by the couple’s intention for childbearing and their concept of a desired family size. Education, access to health care services, awareness, and authority to decide on the number of children have important role in fertility preferences (4, 8-10). Evidences suggest that in many parts of the world, fundamental change has taken place in an individual’s attitude towards marriage and childbearing (2).

One of the main issues in the field of fertility and in assessing the factors related to fertility behavior is the couple’s preferences-fertility and the desire to bearing child. Aside from this, the number of desired children is considered a very important and serious issue in predicting the actual number of children that couple would desire as emphasized by various studies conducted by Habbema et al. (11), and Günther and Harttgen (12). After three years of policymaking on the population of Iran, few studies have been conducted specially on childbearing trends. This study aimed to determine desired number of children, fertility preference, and related factors among people refer to pre-marriage counseling to clarify their presumed behavior in case of fertility.

## Materials and Methods

The present descriptive analytic cross sectional study was conducted in the Province of Alborz, located in the neighboring capital of Iran (with a distance of 45 km) having an area of 5121 square kilometers and a population of about 3 million, between May 2014 and March 2015. This province, due to its nearness to Tehran is the reason behind the number of migration taking place in different parts of the country and due to the diversity of ethnicity has been coined a name “small Iran”. The study populations were women and men who planned to marry in near future. The mentioned couples referred to the pre-marriage counseling centers of Alborz province. These centers are two university pre-marriage counseling centers that one of them is referral.

This paper was a part of the larger study with 60 variables (items) and we have selected 10 samples for each variable. As a result, 600 women and men were recruited. Based on convenience sampling method, during 8 months all the women and men who came to two pre-marriage counseling centers including 300 couples (600 individual) aged between 15 to 45 years participated in this study and were asked to complete a questionnaire. Exclusion criteria were lack of consent to participate in the study or any other nationality except being Iranian. A written informed consent was obtained from each participant after explaining the aims of the study. None of the participants were excluded. The self-administered anonymous questionnaire was included 13 items; 7 items about demographic characteristics, 6 items related to participants’ interest and preference related to fertility. The questionnaire included variables such as: age, education, occupation, condition of house, predicted status of future residence, monthly family income, number of brother (s) or sister (s), desired number of children, number of children based on the child’s gender, suitable years of interval for conception of the first baby after marriage, suitable age of marriage for females, suitable age of marriage for males, level of interest to become a father/mother and suitable years of interval between pregnancies.

This questionnaire was designed and implemented by the Ministry of Health and Medical Education, Tehran throughout the country in order to assess the decision making patterns of childbearing intentions. Validity and reliability of the questionnaire was evaluated. Content validity was analyzed by experts in the field of fertility and demographics and minor corrections implemented. Reliability of the questionnaire was determined as 0.88. Statistical analysis was done using SPSS software version 19. Also descriptive and analytical statistics were implemented to data analysis. This study was a small part of a larger study designed by MOH of Iran. Ethic Committee of Alborz University of Medical Sciences approved the study (ABZUMS.Rec.1393.45).

## Results

The age range of male participants was between 19-54 years (mean ± SD: 28.36 ± 5.8) while the in female participants was between 13-45 years (mean ± SD: 24.8 ± 5.9). Majority of the males were between the 20-30 years of age (66.6%) while females were below 25 years of age (57%). A higher percentage of men than women had education level less than diploma. A large percentage of men (87%) were employed, while only 35% of women employed. Both, men and women thought that they are going to live in a rental house. About half of the male participants and 37.3% of females earned less than 10 million Rials (280 US $) respectively. The vast majority of the participants were anticipated that they will live in a house with size of 50 to 100 square meters. More than 70% of samples had 1 to 4 siblings (Table 1).

In item of the participants’ desire to be parent, more than 80% of men and 70% of women stated that they are interested much and/or very much to be parent. Less than 2 percent of both genders were not interested to be parent. The male participants believe that appropriate age for marriage is 26.3 for men and 21.6 for women and the female participants mention 27.2 and 22.9 respectively for men and women. Appropriate time for attempting the first conception after marriage was 2.7 years from opinions of men and women. About 17 percent of men and 22.3 percent of women stated that they want to have 1 child and equally 52.7 percent of men and women wanted to have 2 children. The only factor that contributed to the female participant’s decision for a desirable number of children was the number of siblings that they have. There was a convergence in desired number of children in male and female participants. About 10% of men and 11% of women want to have 2 children no difference in gender. Three percent of men and none of the women have decided to have no child. Demographic characteristics and desired number of children according to gender were analyzed. Bivariate test showed that there was significant difference between numbers of women’s sibling and desired numbers of children (P<0.001). Other demographic characteristics were not related to desired numbers of children. With the increase in the number of siblings, desired numbers of children was increased.

**Table 1:** Demographic characteristic of participants


Variable		Men		Women		Total	
		n	%	n	%	n	%

Age							
	<20	12	4	83	27.3	95	15.8
	20-24	88	29.3	88	29.3	176	29.3
	25-39	112	37.3	78	26	190	31.7
	30-34	56	18.7	36	12	92	15.3
	35-39	20	6.7	11	3.7	31	5.2
	40-44	7	2.3	4	1.3	11	1.8
	≥45	5	1.7	0	0	5	0.9
Education							
	Less than diploma	169	56.3	148	49.3	317	52.8
	Diploma and higher	131	43.7	152	50.7	283	47.1
Job							
	Employee	261	87	105	35	366	61
	Unemployed	21	7	170	56.0	191	31.8
	Job seeker	18	6	25	8.4	43	7.2
Opinion about housing condition in future							
	Renter	139	46.4	120	40	259	43
	Landlord	106	35.3	111	37	217	36
	Belong to father	55	18.3	69	23	124	21
Income							
	No income	7	2.3	138	46	145	24.2
	< 1 million tomans	148	49.3	114	37.3	262	43.6
	1 to 2 million tomans	107	35.7	41	13.7	148	24.7
	2 to 3 million tomans	26	8.7	5	1.7	31	5.2
	> 3 million tomans	12	4	2	0.7	14	2.3
Opinion about house size in future							
	<50 m^2^	15	5	9	3	24	4
	51-75 m^2^	113	37.3	108	36	221	36.8
	76-100 m^2^	113	37.7	119	39.7	232	38.7
	101-150 m^2^	40	13.3	56	18.6	96	16
	>151 m^2^	19	6.3	8	2.7	27	4.5
Number of siblings							
	None	10	3.3	19	6.3	29	4.8
	1 to 4	213	71	231	77	444	74
	5 to 8	67	22.3	56	15	112	18.7
	> 8	10	3.3	5	1.7	15	2.5


Table 2 shows demographic characteristic and the first birth interval according to gender. There was a statistically significant relationship between the age of marriage and the first birth interval (P<0.001). Also education (P<0.001) and numbers of siblings of the participants (P=0.007) were related to the first birth interval. With the increase in marriage age and education level, the first birth interval was increased, but having more siblings led to decrease in the first birth interval marriage age, education, income and numbers of women’s siblings were related to proper age for male marriage (Table 3). These relationships were positive for all mentioned variables. The same variables were related to proper age of male marriage that had relationship to proper age of female marriage (Table 4). There was a statistically significant relationship between income (P=0.01), house size of male participants (P=0.01) and pregnancy intervals (Table 5). Increasing the number of siblings was associated with pregnancy intervals inversely (P<0.05). In term of interest to be parents, there was a significant relationship with ages of the male participants (P=0.01). Indeed older men had lower interest to be parent (Table 6).

**Table 2:** Demographic characteristic and the first birth interval according to gender


Demographic variable	Gender	The first birth interval	P value

Marriage age	Male	Krus-kalwalis	<0.001^*^
	Female	Krus-kalwalis	<0.001^*^
Education	Male	Chi-square	<0.001^*^
	Female	Chi-square	<0.001^*^
Job	Male	Chi-square	0.2
	Female	Chi-square	0.91
Housing condition	Male	Chi-square	0.07
	Female	Chi-square	0.47
Income	Male	Chi-square	0.19
	Female	Chi-square	0.4
House size	Male	Chi-square	0.4
	Female	Chi-square	0.22
Number of sisters and brothers	Male	Spearman	0.007^*^
	Female	Spearman	0.01^*^


*; Significant at P<0.05.

**Table 3:** Demographic characteristic and proper age for male marriage according to gender


Demographic variable	Gender	Proper age for male marriage	P value

Marriage age	Male	Spearman	<0.001^*^
	Female	Spearman	<0.001^*^
Education	Male	Mann-withney	0.001^*^
	Female	Mann-withney	0.002^*^
Job	Male	Mann-withney	0.88
	Female	Mann-withney	0.19
Housing condition	Male	Krus-kalwalis	0.001^*^
	Female	Krus-kalwalis	0.19
Income	Male	Krus-kalwalis	0.001^*^
	Female	Krus-kalwalis	0.005^*^
House size	Male	Mann-withney	0.31
	Female	Mann-withney	0.43
Number of sisters and brothers	Male	Spearman	0.37
	Female	Spearman	0.004^*^


*; Significant at P<0.05.

**Table 4:** Demographic characteristic and proper age for female marriage according to gender


Demographic variable	Gender	Proper age for female marriage	P value

Marriage age	Male	Spearman	<0.001^*^
	Female	Spearman	<0.001^*^
Education	Male	Mann-withney	<0.001^*^
	Female	Mann-withney	<0.001^*^
Job	Male	Mann-withney	0.62
	Female	Mann-withney	0.4
Housing condition	Male	Krus-kalwalis	0.01^*^
	Female	Krus-kalwalis	0.53
Income	Male	Krus-kalwalis	0.01^*^
	Female	Krus-kalwalis	0.001^*^
House size	Male	Mann-withney	0.68
	Female	Mann-withney	0.54
Number of sisters and brothers	Male	Pearson	0.05
	Female	Pearson	0.001^*^


*; Significant at P<0.05.

**Table 5:** Demographic characteristic and pregnancy intervals according to gender


Demographic variable	Gender	Pregnancy intervals	P value

Marriage age	Male	Krus-kalwalis	0.06
	Female	Krus-kalwalis	0.11
Education	Male	Chi-square	0.19
	Female	Chi-square	0.3
Job	Male	Chi-square	0.68
	Female	Chi-square	0.3
Housing condition	Male	Chi-square	0.62
	Female	Chi-square	0.28
Income	Male	Chi-square	0.01^*^
	Female	Chi-square	0.47
House size	Male	Chi-square	0.01^*^
	Female	Chi-square	0.6
Number of sisters and brothers	Male	Pearson	0.007^*^
	Female	Pearson	0.01^*^


*; Significant at P<0.05.

**Table 6:** Demographic characteristic and interested to be parents according to gender


Demographic variable	Gender	Interested to be parents	P value

Marriage age	Male	Krus-kalwalis	0.01^*^
	Female	Krus-kalwalis	0.23
Education	Male	Fishers exact test	0.53
	Female	Fishers exact test	0.64
Job	Male	Fishers exact test	0.87
	Female	Fishers exact test	0.33
Housing condition	Male	Fishers exact test	0.07
	Female	Fishers exact test	0.8
Income	Male	Fishers exact test	0.9
	Female	Fishers exact test	0.77
House size	Male	Fishers exact test	0.22
	Female	Fishers exact test	0.36
Number of sisters and brothers	Male	Krus-kalwalis	0.28
	Female	Fishers exact test	0.04^*^


*; Significant at P<0.05.

The only factor that contributed to the female participant’s decision for a desirable number of siblings was associated with the number of siblings that they have. In male participants, no association was observed between the demographic information and their desire in a number of children. With an increasing age at marriage and aspiration for higher educational level, the time interval between marriage and the birth of the first child has increased. But this gap has decreased with the increasing number of siblings. With increasing age of marriage, education, income and type of residence, male participants have believed that the age of marriage of the male gender should be higher. In female participants, age of marriage, education, income, and the number of sister(s) or brother (s) were associated to appropriate age of men for marriage.

The same relationship exists where women considered a certain age to be suitable for marriage. With the increase in men’s income, the preference for bigger houses and higher intervals for pregnancies have increased but with the increasing number of siblings both female and male groups stated less number of intervals between desired pregnancies. With the increasing age of men at marriage, the desire to become a father has decreased but this issue has not been observed in women. With the increasing number of siblings in women, their desire to become a mother has increased but this case yield an opposite results for male participants. There exist an association among an increasing desire for parenthood, increasing number of desired children and also a lesser interval between marriage and the first conception. For fathers, this desire was associated with a desire to decrease the interval between pregnancies.

## Discussion

In this study, we presented viewpoints of young Iranian people and explored related factors. About 50% of the female and male participants have expressed that the desired number of children for a desired family is 2 (one girl, one boy). In this study, the desired number of children per woman from the perspective of the participants was calculated to be approximately 1.91 which is below replacement level. Some sociologists and policy makers strongly believed that fertility rate is mostly influenced by the demands and preferences of families especially women, regarding the desired number of children. Also the study conducted by Günther and Harttgen (12) indicated a strong correlation between the desired number of children and the actual number of children. The desired number of children per generation could be affected by the context in which an individual grows (9). Although some studies have not confirmed this issue, the actual number of children for a family to the desired number can be completely different (13). They believed that between the number of children expected, and the number of actual children, there was a slight difference in developed and developing countries. Developing countries with a high fertility rate usually expects a lower number of children, lower than the actual number but in developed countries the opposite is true. In a study conducted in Japan, the actual number of children was 1.77 and the number of desired one has been reported 2.55 which is significantly lower than the number of children considered desired (7). The desired number of children not necessarily will be concordant with the reality of fertility behavior of families that named "fertility gap". The reason of this gap can be due to socio-economic factors such as divorce, financial problems, higher education, employment and aspiration for higher income (4).

The number of desired children as expressed by 83.1% of the participants in this study was between 1 to 2. About 20 % favors one child while 63.3% favors 2 children. On the other hand, despite the immense interest of the couple to become parents as observed in this study the desire for less conception can serve as a warning sign for an increasing decline of fertility in the country resulting to an aging population and a reduction of the productive younger generation. It seems that the overall population policies must be directed towards more favorable conditions for economic security and welfare for the society especially the women’s need in nurturing her child and transform these policies to the stage of implementation in order to increase fertility to a satisfactory condition.

Although in some western countries the desire to be childless is an ordinary issue, studies have indicated that European and especially Asian countries desire to have the number children of that everyone wants which, is the foundation of living (3, 7). In Japan, 58.4% of individuals aging 20-29 years old have expressed that 2 children is desired for a family, in South Korea 58.7, in the United States of America 42.2% and in France 56.6% have expressed 2 children as a desired number in a family (14). Interestingly, there exist a similar tendency in the desired number of children in most countries and it seems that there is a convergence of opinions in this issue in most developed countries and also in Iran.

In the present study, none of the demographic factors were related to the men’s choice for a desired number of children while in women, the increased number of siblings has been reported to affect the choice for a desired number. In some studies, the relationship between age at marriage, education, income and financial situation justifies childbearing behavior (8, 13). In fact, motivation to higher education and higher incomes decreases the motivation for childbearing (4, 15). The result of this study about relation between the participants’ age at marriage and desired number of children was in line with that of another study in Iran (5).

The results of a study conducted to assess fertility desire of women in Tehran showed that poor income was related to fertility disinterest (16). Although some researchers argued that income might not purely interpret child birth behaviors of couples, it is necessary to assess the different kinds of social support that people receive (10). One related reason that higher education levels may lead to less childbearing is balancing between education affairs and mother roles. Moreover, more- educated women may attain to a better career than other women. Also more income and authority, may provide more control on childbearing for educated women (17). The possible reason for the difference in the results of the present study in comparison to other studies might be due to the fact that only 12.7% of male participants have monthly income of 600 US dollars and approximately 50% have an income of 300$ monthly. In women, these figures have been less. An important point in this study is that there is a unanimous agreement among individuals with different demographic characteristics in the desired number of children a couple should have.

In this study, advanced age of men and women during marriage and the pursuit of higher education has increased the interval between marriage and the conception of the first child and also the increased age considered appropriate for marriage in men and women. With improved income, the age considered appropriate for marriage in both genders have also increased. Findings in this study correspond to the findings of the study conducted by Ericsson et al. (18) in Sweden which is a qualitative study on professional men and women postponing conception in favor to acquire higher education. Participants expressed that the reason for delayed childbearing in men and women having higher education is to cope with adapting social changes and the new life style. Hence; a change in priorities. Another reason is that nowadays children are expensive and then parents decide to postpone their childbearing until they feel they achieve to more stable economic position. Therefore it is predictable that most of the couples will have their first child at later age. Advanced maternal age can pose a lot of problems in considering the sociocultural changes in Iran’s present society in which a little less than half of the female population have university education. With increasing age of marriage and time interval between marriage and conception of the first child, decline in female fertility will occur (19). Furthermore; advanced maternal age during pregnancy may be associated with more medical and obstetrical complications for mother and fetus that could create a negative effect on population growth, health and dynamics respectively (20-22).

Accordingly; as indicated by results of some studies conducted on Iranian society, there is an increasing trend between the interval of marriage and the conception of the first child which can generally affect the rate of fertility (2). Some studies have shown that there were misconceptions regarding fertility in a way that couples presumed that with the emergence of the modern methods of fertility treatments, age associated infertility problems will be completely resolved. Based on the conducted by Virtala et al. (23) more than 50% of male and 1/3 of female college students have believed that decline in fertility would only occur at the age of 45 in women. Also, in another study conducted on non-medical students, they believed that women’s fertility can still be preserved even with increasing age. Therefore, they can plan to have their pregnancy at ages when fertility declines (24).

Community policy-makers must be aware of this issue and should address significant issues in their policies regarding childbearing to support families. Some studies have shown that policies designed and implemented to resolve conflict between work and study have played an important role in strengthening the couples desire for childbearing and helping couples to counter their decision for postponement of conception on their first child (4). Although the availability of contraceptive methods is considered to be one of the factors affecting reproductive behavior, the most important factor in order to achieve success in changing the community’s behavior towards fertility is more understanding in order to reduce conflicts between maternal and paternal roles, education and employment (4).

In this study, having more siblings was related to higher number of desired children in women, the shorter first birth intervals, and pregnancy intervals in men and women. One key point in this regard is the effect that someone may receive from his family background variables. It may be related to common values of individuals in a family. Several studies have emphasized the essential role of social interaction for fertility behaviors (4, 25). The present study has several strengths. It was a part of the first national study about fertility preferences and desired numbers of children. The mentioned topic that has also not been studied among the general population in Iran. Convenience sampling was a limitation of our study. The sample was a part of the large study that has been conducted by MOH of Iran. The response rate was very good and neat to all of the couples answered to the questionnaire, therefore the study results reflect their opinions reliably. Further studies with a larger number of samples, or nationally representative studies are suggested to achieve to more precise findings. In addition, future studies are proposed to assess attitude of couples about fertility and their fertility awareness to obtain more interpretable findings.

## Conclusion

In this study, preferences and desires related to the reproductive behavior on couple’s prior to marriage were evaluated and one of the strong points that can be pointed out is that, these couples will serve as representatives for the whole province for the reason that all couples would refer to these 2 clinics for pre-marriage counseling. Majority of the participants of the present study express their desire to have one or two siblings. In considering the fact that what one desires does not always come into reality, the risk of reduced fertility is generally present in the community and in order to create a favorable environment for childbearing, appropriate policies should be implemented.

## References

[1] Abbasi-Shavazi M, McDonald P (2006). The fertility decline in the Islamic Republic of Iran, 1972- 2000. Asian Popul Stud.

[2] Abbasi-Shavazi MJ, Morgan SP, Hossein-Chavoshi M, McDonald P (2009). Family change and continuity in iran: birth control use before first pregnancy. J Marriage Fam.

[3] Peterson BD, Pirritano M, Tucker L, Lampic C (2012). Fertility awareness and parenting attitudes among American male and female undergraduate university students. Hum Reprod.

[4] Mills M, Rindfuss RR, McDonald P, te Velde E (2011). ESHRE Reproduction and Society Task Force.Why do people postpone parenthood? Reasons and social policy incentives. Hum Reprod Update.

[5] Shiri T, Bidarian S (2009). Economic factors affecting the fertility of women 15-49 years old population employed in the school districs 22 of Tehran. Journal of Social Sciences.

[6] Ding QJ, Hesketh T (2006). Family size, fertility preferences, and sex ratio in China in the era of the one child family policy: results from national family planning and reproductive health survey. BMJ.

[7] Matsumoto Y, Yamabe S (2013). Family size preference and factors affecting the fertility rate in Hyogo, Japan. Reprod Health.

[8] Pham DT, Stephens EH, Antonoff MB, Colson YL, Dildy GA, Gaur P (2014). Birth trends and factors affecting childbearing among thoracic surgeons. Ann Thorac Surg.

[9] Berrington A, Pattaro S (2014). Educational differences in fertility desires, intentions and behaviour: a life course perspective. Adv Life Course Res.

[10] Rindfuss RR, Guilkey D, Morgan SP, Kravdal O, Guzzo KB (2007). Child care availability and first-birth timing in Norway. Demography.

[11] Habbema JD, Eijkemans MJ, Leridon H, te Velde ER (2015). Realizing a desired family size: when should couples start?. Hum Reprod.

[12] Günther I, Harttgen K (2016). Desired fertility and number of children born across time and space. Demography.

[13] Goldstein J, Lutz W, Testa MR (2003). The emergence of sub-replacement family size ideals in Europe. Popul Res Policy Rev.

[14] Morita M, Ohtsuki H, Sasaki A, Hiraiwa-Hasegawa M (2012). Factors affecting the number of children in five developed countries: a statistical analysis with an evolutionary perspective. Letters on Evolutionary Behavioral Science.

[15] Kravdal Ø (2002). Education and fertility in sub-Saharan Africa: individual and community effects. Demography.

[16] Tavousi M, Motlagh ME, Eslami M, Haerimehrizi A, Hashemi A, Montazeri A (2015). Fertility desire and its correlates: a pilot study among married citizens living in Tehran, Iran. Payesh.

[17] Amuedo-Dorantes C, Kimmel J (2005). The motherhood wage gap for women in the United States: the importance of college and fertility delay. Rev Econ Househ.

[18] Eriksson C, Larsson M, Skoog Svanberg A, Tydén T (2013). Reflections on fertility and postponed parenthood-interviews with highly educated women and men without children in Sweden. Ups J Med Sci.

[19] Yoldemir T (2016). Fertility in midlife women. Climacteric.

[20] Carolan M, Frankowska D (2011). Advanced maternal age and adverse perinatal outcome: a review of the evidence. Midwifery.

[21] Kenny LC, Lavender T, McNamee R, O’Neill SM, Mills T, Khashan AS (2013). Advanced maternal age and adverse pregnancy outcome: evidence from a large contemporary cohort. PLoS One.

[22] Balasch J, Gratacós E (2012). Delayed childbearing: effects on fertility and the outcome of pregnancy. Curr Opin Obstet Gynecol.

[23] Virtala A, Vilska S, Huttunen T, Kunttu K (2011). Childbearing, the desire to have children, and awareness about the impact of age on female fertility among Finnish university students. Eur J Contracept Reprod Health Care.

[24] Sahib Khalil N, Israa TH, Hussam DS (2015). Fertility awareness among medical and non- medical undergraduate university students in Al-Iraqia University, Baghdad, Iraq. American Journal of Medical Sciences and Medicine.

[25] Regnier-Loilier A (2006). Influence of Own sibship size on the number of children desired at various times of life: the case of France. Population.

